# Making the Thermodynamic Cost of Active Inference Explicit

**DOI:** 10.3390/e26080622

**Published:** 2024-07-24

**Authors:** Chris Fields, Adam Goldstein, Lars Sandved-Smith

**Affiliations:** 1 Independent Researcher, 11160 Caunes Minervois, France; 2 Department of Physiology, Anatomy and Genetics, University of Oxford, Oxford OX1 3PT, UK; adamjuliangoldstein@gmail.com; 3 Monash Centre for Consciousness and Contemplative Studies, Monash University, Melbourne 3168, Australia; lars.sandvedsmith@gmail.com

**Keywords:** compartmentalization, control flow, Free Energy Principle, matrix representation, mortal computation

## Abstract

When describing Active Inference Agents (AIAs), the term “energy” can have two distinct meanings. One is the energy that is utilized by the AIA (e.g., electrical energy or chemical energy). The second meaning is so-called Variational Free Energy (VFE), a statistical quantity which provides an upper bound on surprisal. In this paper, we develop an account of the former quantity—the Thermodynamic Free Energy (TFE)—and its relationship with the latter. We highlight the necessary tradeoffs between these two in a generic, quantum information-theoretic formulation, and the macroscopic consequences of those tradeoffs for the ways that organisms approach their environments. By making this tradeoff explicit, we provide a theoretical basis for the different metabolic strategies that organisms from plants to predators use to survive.

## 1. Introduction

Active Inference Agents (AIAs) are physical systems compliant with the Free Energy Principle (FEP); such systems maximize their abilities to predict the behaviors of their environments by learning from experience and by actively probing their environments to gain new information [1,2,3,4,5]. Whether they are bacteria, humans, robots, or simulations running on ordinary computers, AIAs need adequate supplies of thermodynamic free energy (TFE)—in biological systems, metabolic energy [6]—to power interactions with their environments. It is, in particular, the flux of TFE through an AIA, entering as “fuel” and exiting as “waste” heat, that powers autopoiesis and hence maintains the AIA as a dissipative system, preventing thermal equilibration with its environment. The need of any AIA to maintain its TFE supply solves the “dark-room problem” posed by the goal of uncertainty minimization; no AIA can minimize uncertainty simply by minimizing its environmental input due to the pain of starving to death [7].

Sengupta, Stemmler, and Friston [8] showed in 2013 that any AIA minimizes the TFE requirements of its computational processes when it minimizes prediction errors, i.e., when it minimizes the variational free energy (VFE) at its boundary. The reason is straightforward: when predictions are accurate, incoming data do not induce computationally expensive state changes. Formal treatments of active inference under the FEP have, therefore, subsequently focused on the minimization of VFE, treating TFE inputs such as food just as “preferred” observational outcomes [6,9,10,11]. An exclusive focus on VFE, however, makes it difficult to distinguish two types of problem solving: activity with the specific goal of obtaining TFE resources and activity that has other goals. This, in turn, makes it difficult to explicitly address any specific role of TFE limitations in modulating attention, problem solving, or planning. We know, however, that organisms employ such TFE-driven modulatory processes, and we can expect “mortal computers” [6] to do so as well.

Here, we develop an explicit representation of TFE inputs, or dually waste heat outputs, within the formalism of the FEP, and ask how active management of TFE resources contributes to control flow during computation. We begin in Section 2 by noting that the FEP can be regarded either as “just physics” or as a theory of inference, particularly as a theory of approximate Bayesian VFE minimization. We distinguish between models that adopt either of these stances and models that attribute “inference” or “cognition” to only some components of a system while treating the rest of the system as infrastructure. Building on previous work [12,13], we show in Section 3 that models of the latter kind require an explicit representation of TFE flow for completeness. We then consider in Section 4 how the active allocation of TFE flows to component processes provides a control mechanism for compartmentalized multi-component systems. Finally, in Section 5, we address the practical issue of estimating TFE flows as they affect information processing within the discrete matrix formulation of active inference developed in [14].

## 2. “Just Physics” versus Implemented Computation

We consider finite system *S* that interacts with a finite environment *E* and assume that the joint system U=SE is effectively isolated. The FEP characterizes the conditions under which *S* and *E* remain distinguishable from each other as the joint system *U* evolves through time. It states, speaking informally, that *S* and *E* remain distinct only if they are only sparsely or weakly coupled [9]. This condition can be formulated in various ways; one can require that almost all paths through the joint space that begin in *S*(*E*) remain in *S*(*E*) [11], that the number of states on the Markov blanket (MB) between *S* and *E* be much smaller than the number of states in either *S* or *E*, or that the interaction Hamiltonian (or total interaction energy operator) $H_{SE}$ be much smaller than either of the self-interactions $H_{S}$ and $H_{E}$ [13]. What all of these conditions assure is that both *S* and *E* have “internal states” that are not directly involved in the interaction and that therefore remain mutually conditionally statistically independent. These internal states can then implement distinct, independent computations that enable *S* and *E* to exhibit distinct, agentive behaviors.

The FEP is, therefore, fundamentally a principle about physical interaction, and hence about the exchange of energy between physical systems. It becomes a principle about inference when energy flow is interpreted as information flow. This interpretation rests on Clausius’ [15] definition of entropy dE=TdS, where E is energy, *T* is ambient temperature, and S is entropy, and on Boltzmann’s [16] identification of entropy with uncertainty about the state of a system, $S=k_{B}\ln\Omega$, where $k_{B}$ is Boltzmann’s constant and Ω is the number of observationally indistinguishable states of the system of interest. Combining these two yields Landauer’s principle, $dE=\ln2(k_{B}T)$ for the minimal energy dE required to resolve the value of one bit, i.e., to resolve the state of a two-state system [17,18]. Any energy flow, therefore, can be associated with a maximal number of bits, and hence with a maximal information bandwidth. With this information-flow interpretation of energetic coupling, the FEP becomes the claim that the input/output (I/O) bandwidths of persistent systems are small compared to the internal information flows—computations—that generate outputs given inputs. Persistent systems, in other words, remain persistent by implementing computations that effectively model the observable behavior their environments and acting accordingly, i.e., by being AIAs.

**Figure 1:**
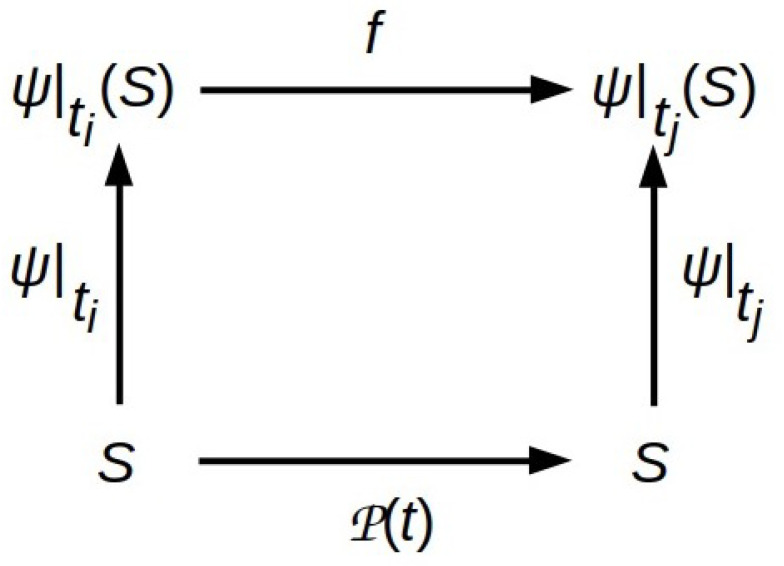
Generic structure of semantic interpretations of physical processes. Function *f* interprets, via the semantic map ψ, the action of the physical time-propagator P(t) between time points $t_{i}$ and $t_{j}$. The interpretation is semantically coherent provided the diagram commutes, i.e., provided ${f|}_{t_{i}\to t_{j}}\left(\psi {|}_{t_{i}}\left(S\right)\right){=\psi |}_{t_{j}}\left(P{|}_{t_{i}\to t_{j}}\left(S\right)\right)$.

The idea that arbitrary physical systems can be interpreted as information-processing systems—computers—is not unique to the literature of the FEP; indeed, it is ubiquitous in physics [19] and forms the basis for explanation by appeal to function in the life sciences [20] and computer science [21]. The structure of any such interpretation is shown in Figure 1. The vertical map ψ is *semantic* in Tarski’s model-theoretic sense [22]: it treats function *f* as *implemented by* physical process P(t) between time points $t_{i}$ and $t_{j}$. As Horsman et al. point out, such semantic maps can also be thought of as representing measurements [19]; in this case, Figure 1 depicts the relationship between any observation-based model *f* and the physical process P(t) that it models.

Representing physical systems as AIAs employs the mapping process shown in Figure 1: the physical system behaves “as if” it is executing inferential processes encoded by some function *f* that construct a model of its environment’s behavior and then employ that model to choose approximately Bayes-optimal actions. This inferential process must satisfy two constraints: (1) its only inputs from the environment are the data encoded on its MB; and (2) it must be tractable. As emphasized in [8,9] and elsewhere, these constraints are met optimally by function *f* that minimizes an upper bound on the surprise −lnp(b|η), where *b* is an input “sensory” MB state and η is a model prediction. This upper bound is the VFE ([9] Equation (2.3)),

(1) $$F=D_{KL}\left[q_{\mu }\left(\eta \right)|p\left(\eta \right)\right]-E_{q}\left[\ln p\left(b|\eta \right)\right],$$

where $q_{\mu }\left(\eta \right)$ is a variational density over predicted external states η parameterized by internal states μ and $E_{q}$ is an expectation value operator parameterized by variational density *q*.

We can therefore choose to regard an AIA simply as a dissipative physical system that is maintaining its state in the vicinity of—or maintaining an orbit around—a nonequilibrium steady state (NESS), or we can choose to regard it as computer implementing a procedure that minimizes an abstract information measure, the VFE defined by Equation (1). Provided that states *b* are sampled from the complete state space of the MB separating the system from its environment—and hence capture the total energy/information exchange through the MB—descriptions of the dynamics as “just physics” or “just computations” are related by semantic map ψ as in Figure 1. The energy and information flows they entail are, at optimal thermodynamic efficiency, quantitatively related by the total I/O bandwidth of the MB in bits times ln2(*k_B_T*).

In practice, however, we do not always want to view systems as either “just physics” or “just computation”. We often want to view part of a system as computing some specific function, and the rest as providing the infrastructure services required by the system’s physical embodiment, including architectural integrity, adequate power, and heat dissipation. We are in this situation whenever specific computations are attributed to particular components of system *S*, or when only a particular subset of *S*’s MB states is regarded as encoding “interesting” inputs and outputs. Note that this choice of what is “of interest” is effectively a choice of semantic map ψ that applies to only some components of *S*. This kind of interest-driven decomposition is ubiquitous in biology, e.g., when distinguishing signal transduction from metabolic pathways in cells, when modeling neural computation in terms of synaptic inputs and outputs, or when treating the I/O of animal’s brain as separate and distinct from that of its digestive system. It is also ubiquitous in practical computing, e.g., when specifying the application programming interface (API) of a software module while leaving power management to the hardware and memory management to the operating system.

Interpreting particular subsystems of system *S* as computing particular functions abstracts away the fundamental constraints imposed on *S* by its physicality, including the fact that acting on the environment by producing output requires TFE in accord with Landauer’s principle. Given the assumption that U=SE is isolated, that energy must be obtained from the environment as an input. Providing a complete description of an AIA that computes some specific inputs and outputs—or sensations and actions—of interest requires, therefore, also the thermodynamic (or metabolic) inputs and outputs that the “of interest” designation assumes as infrastructure. It therefore requires devoting some of the states on the MB to flows of fuel and waste heat. Making these requirements of physical embodiment explicit, thus re-integrating thinking about software with thinking about hard- or bio-ware, is one of the goals of both the embodied cognition and mortal computing frameworks [6].

## 3. Coupling Information and Energy Flows

If computational and infrastructure functions are regarded as performed by distinct components of a system, how do we represent their coupling? In the notation of Figure 1, if we factor the interpretation of ψ, what is the relationship between the factors? How is TFE delivered to the computational processes that need it in order to compute VFE?

This question is challenging to formulate precisely, because any decomposition of system *S* into components generates an MB between them and renders each component a part of the environment of each of the others. Decomposition requires, therefore, a bottom level of undecomposed “atomic” components to avoid infinite regress. At this atomic level, the question of how computing and infrastructure relate must be answered without recourse to further decomposition.

This question of how “physical” TFE flows couple to “computational” VFE flows arises in both classical and quantum formulations of the FEP. It is, however, most easily addressed using quantum formalism, which provides a simple, intuitive description of inter-system interactions that applies to all systems, regardless of their structure. Using this formalism, we can view TFE and VFE flows as distinguished by a symmetry breaking that has no natural classical formulation [23]. We first review the quantum formulation of generic physical interactions, then show how it provides both a natural definition of “atomic” systems and a precise characterization of the interaction between components in a composite system. We use the latter to understand how a thermodynamic component, effectively power supply, can provide regulated TFE flows to computational components of a composite system.

In quantum formalism, the joint state space of a composite system U=SE is a finite-dimensional Hilbert space $H_{U}=H_{S}⊗H_{E}$ [13,24]. For any system *X*, the Hilbert space $H_{X}$ is a vector space that can be constructed by assigning a basis vector to every independent yes/no question that can be asked about system *X*. Each of these basis vectors can be represented by a quantum bit, a qubit, with measurable states (in the Dirac notation) | ↑〉 and | ↓〉. Hilbert spaces $H_{U},H_{S}$, and $H_{E}$ can, therefore, all be considered qubit spaces; see [25] for a textbook introduction to such spaces. We let B denote the boundary between *S* and *E* implicitly given by factorization $H_{U}=H_{S}⊗H_{E}$. Systems *S* and *E* can be considered distinct only if they have distinct, mutually conditionally independent states |S〉 and |E〉. This is the case only if their joint state is separable; i.e., only if it factors as |SE〉=|S〉|E〉. In this case, the entanglement entropy across B is zero. The FEP, in this formulation, states the truism that distinguishable systems must remain unentangled.

The interaction between *S* and *E* is represented in quantum formalism by a Hamiltonian or total energy operator $H_{SE}$. This operator is linear, and so it can be written as $H_{SE}=H_{U}-\left(H_{S}+H_{E}\right)$, where $H_{U}$, $H_{S}$, and $H_{E}$ are the internal or “self” interactions of *U*, *S*, and *E*, respectively. Interaction $H_{SE}$ is defined at boundary B. We can characterize both $H_{SE}$ and B by employing the Holographic Principle [26,27] which states that the information that can be obtained about any system *X* by an observer outside *X* is limited to the information that crosses boundary $B_{X}$ of *X*. If *X* is finite, this quantity of information is finite, and can be written as classical entropy $S(B_{X})$. We can therefore think of boundary B between *S* and *E* as encoding S(B)=N qubits, and hence as characterized by an ancillary Hilbert space $H_{B}$ with dimension $\dim\left(H_{B}\right)=\dim\left(H_{SE}\right)=2^{N}$. Hilbert space $H_{B}$ is ancillary because it is not part of U=SE, i.e., $H_{B}\cap H_{U}=\emptyset$. This reflects the fact that B is merely a theoretical construct induced by factorization $H_{U}=H_{S}⊗H_{E}$.

Given this characterization of B, we are now in a position to describe internal dynamics $H_{S}$ of *S*. Formally, $H_{S}$ is a linear operator on state space $H_{S}$, i.e., we can write $H_{S}:H_{S}\to H_{S}$. Because $H_{S}$ is a space of qubits, we can think of $H_{S}$ as an operator acting on qubits to change their states, i.e., as a quantum computation (again see [25] for an introduction). The only information flowing into *S* from the outside, i.e., from *E*, is the information encoded by the *N* qubits composing B; similarly, the only information flowing out of *S* and into *E* must be encoded by these same qubits. Boundary B is therefore the input/output (I/O) interface to *S* and hence to the quantum computation implemented by $H_{S}$.

**Figure 2:**
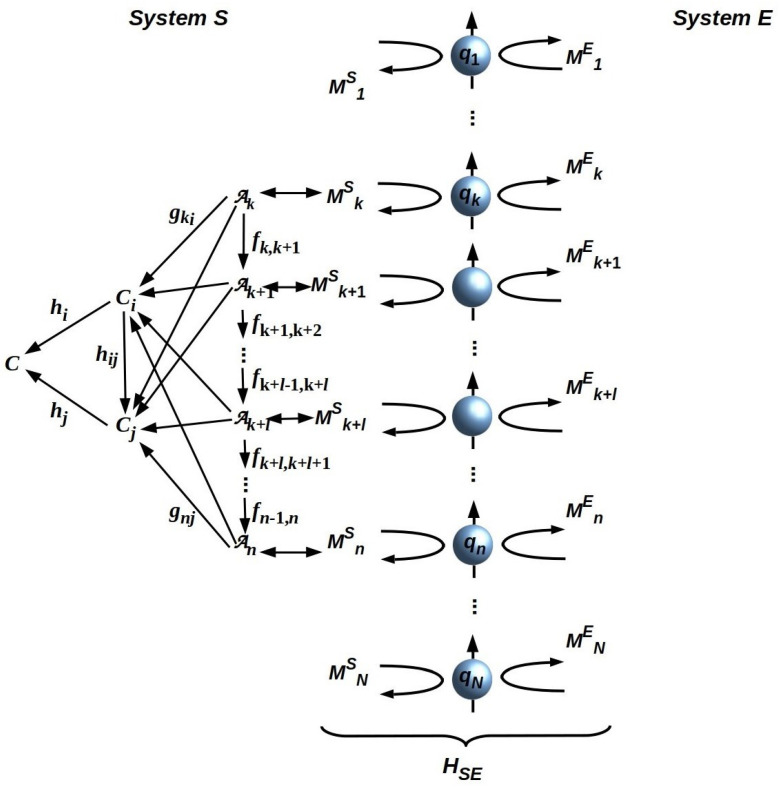
“Attaching” a CCCD to an intersystem boundary B depicted as an ancillary array of qubits. Operators $M_{i}^{k}$, k=S or *E*, are single-bit components of the interaction Hamiltonian $H_{SE}$. The node *C* is both the limit and the colimit of the nodes $A_{i}$; only leftward-going (cocone implementing) arrows are shown for simplicity. See [12,13,24,30] for details. Adapted from [12], CC-BY license.

We can further characterize $H_{S}$ by thinking of B as a finite collection of non-overlapping subsets of qubits, which we call “sectors” $Z_{i}$ and considering the components of $H_{S}$ that act on each of these $Z_{i}$. We can represent each of these components as a quantum reference frame (QRFs) $Q_{i}$ that measures and dually prepares the states of the $n_{i}$ qubits that compose sector $Z_{i}$. A QRF is a physical system that enables measuring or preparing states of other systems in a reproducible way [28,29]; meter sticks, clocks, and the Earth’s gravitational field are canonical examples of laboratory QRFs. Using a QRF such as a meter stick requires, however, implementing a similar QRF internally; an agent that had no internal ability to represent or process information about distances would have no use for a meter stick. Any observer can therefore be considered to implement a collection of QRFs, one for every combination of physical degree of freedom, every physical one observable, that the observer can detect, assign operational meaning to, and process information about [13,24]. Here, we follow previous convention [12,13,24,30] in extending the usual notion of a QRF to include all of the measurement and preparation processes that employ it. As each QRF $Q_{i}$ can also be regarded as a quantum computation, it can also be represented by a hierarchical, communtative diagram—a Cone-CoCone diagram (CCCD)—that depicts information flow between a set of $n_{i}$ single-qubit operators and a single operator $C_{i}$ that encodes an observational outcome for the physical observable represented by $Q_{i}$ [12,13,24,30]. We can depict B and an associated QRF *Q* as in Figure 2.

As mathematical objects, CCCDs are objects in category CCCD; the morphisms of this category are embeddings of small CCCDs into larger ones and projections of small CCCDs out of larger ones [30]; see [31] for a textbook introduction to categories and their uses. Because CCCDs are by definition commutative diagrams, two CCCDs that do not mutually commute cannot be composed to form a larger CCCD. Pairs of non-commuting CCCDs correspond to pairs of non-commuting QRFs, i.e., to pairs of operators $Q_{i}$ and $Q_{j}$ for which commutator $\left[Q_{i},Q_{j}\right]=Q_{i}Q_{j}-Q_{j}Q_{i}\neq 0$. A single quantum process cannot simultaneously implement two non-commuting QRFs. If system *S* implements non-commuting QRFs $Q_{i}$ and $Q_{j}$, it must be partitioned into two subsystems $S_{i}$ and $S_{j}$ that are separated by a boundary via which they interact. Such a system must therefore have distinguishable components, and its components must have different environments. If *E* is the environment of *S*, the environment of $S_{i}$ is $E_{j}=ES_{j}$ and vice versa. Hence, we can define

**Definition** **1.** *An* atomic system *is a system that can be represented as implementing a single QRF.*

Systems that are not atomic are called “composite” systems. The QRFs implemented by an atomic system must, by Definition 1, all mutually commute; composite systems may implement QRFs that do not commute. Note that Definition 1 makes reference to how the system in question is represented. This reflects the fact that an external observer cannot determine what QRF(s) a system implements [32]. How the system is represented is therefore a theoretical choice; indeed, it is the very choice of semantic map ψ that motivates defining atomic systems in the first place.

We let *S* be an atomic system, *E* be its environment, and *Q* be its single (effective) QRF. We can now state the following:

**Theorem** **1.** *The thermodynamic free energy required by an atomic system S is acquired from E via its single (effective) QRF Q.*

**Proof.**  We let $H_{S}$ be the internal dynamics of *S*; by definition, $H_{S}$ implements *Q*. As dom(Q)=B, we can think of *Q* as automorphism Q:B→B (see [30] for details). All TFE required by *S* must traverse B; hence, all TFE required by *S* can only be acquired from *E* via *Q*. □

If we assume that $H_{S}$ is a pure quantum process, and hence that it is perfectly reversible, then it requires TFE only for the thermodynamically irreversible final step of acting on its environment *E*, which we can represent, as in Figure 2, as preparing specific final states of the qubits encoded by its boundary [33,34]. Any additional thermodynamically irreversible steps require additional TFE, up to the limit of fully irreversible classical computation, for which every step requires TFE proportional to the number of bits modified or written. Hence, we can write the TFE consumption of *Q* as

(2) $$\Xi \left(Q\right)=f_{Q}\left(n_{Q}\right)\beta_{Q}k_{B}T_{Q},$$

where $n_{Q}$ is the number of qubits in sector dom(*Q*) on B, $f_{Q}$ is a non-decreasing function with $f_{Q}\left(n_{Q}\right)\geq n_{Q}$ everywhere, $\beta_{Q}\geq \ln2$ is an inverse measure of the thermodynamic efficiency of *Q*, and $T_{Q}$ is the effective ambient temperature. For an atomic system, dom(Q)=B. The minimum value $f_{Q}\left(n_{Q}\right)=n_{Q}$ corresponds to fully reversible computation, i.e., to writing output values on dom(*Q*) as the only thermodynamically irreversible step. For a classical binary tree, $f_{Q}\left(n_{Q}\right)=n_{Q}^{2}\log_{2}n_{Q}$. The value of $\beta_{Q}$ is implementation-dependent, with contemporary semiconductors and ATP/GTP-independent macromolecular switches such as rhodopsins approaching the theoretical optimum, i.e., the Landauer limit of ln2($k_{B}T$) per bit, and ATP/GTP-dependent macromolecular switches typically about 10x less efficient [35].

We now consider system *S* that is atomic and hence has a single QRF *Q* that can be treated as a map Q:B→B. If efficiency $\beta_{Q}$ is fixed, energy $\Xi \left(Q\right)/n_{Q}$ must be obtained from each of the $n_{Q}$ qubits in dom(*Q*). This follows from, and indeed illustrates, a fundamental symmetry of the Hamiltonian $H_{SE}$: permuting the qubits on B, which, since $H_{B}$ is ancillary to $H_{U}$ and just means permuting the labels on $q_{i}$, has no effect on physical interaction $H_{SE}$ [23]. This symmetry is evident from Figure 2, which depicts an atomic system if only qubits $q_{k}\ldots q_{n}$ composing dom(*Q*) are considered. It extends to *Q* itself: since the CCCD representing *Q* is a commutative diagram, permuting the “base-level” operators $A_{i}$ is equivalent to just permuting their labels.

This symmetry of $H_{SE}$ has a significant consequence for computational models of *S*. As Ξ(Q) increases, due to internal irreversibility, i.e., inefficiency, the amount of energy extracted from *E* by the measurement process and dissipated into *E* by the preparation process proportionately increases. Higher-energy interactions disturb *E* more per measurement and inject more noise into *E* per preparation. The symmetry of $H_{SE}$ spreads this increased disturbance and noise uniformly across B.

**Figure 3:**
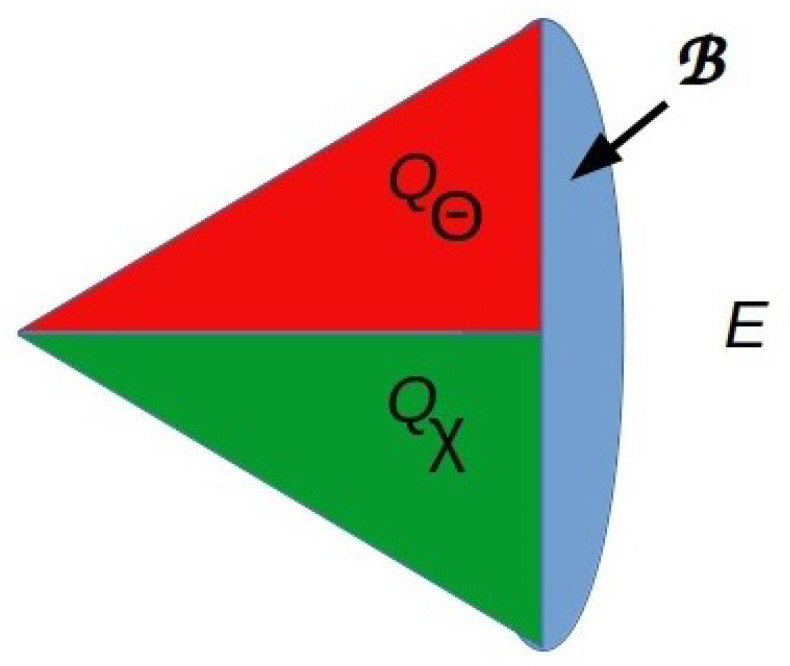
Factoring a QRF *Q* into components $Q_{\chi }$ and $Q_{\Theta }$ allows information exchange through B to be separated from thermal exchange through B. This breaks the previous qubit-exchange symmetry on B as discussed in Ref. [12].

Therefore, from Equation (2), we can see that any system *S*, whether atomic or composite, faces an energetic tradeoff for every deployed QRF *Q*. Systems operating far from the optimal, fully reversible limit of $f_{Q}\left(n_{Q}\right)\beta_{Q}=n_{Q}$ln2 can decrease the interaction energy for measurement and preparation locally by breaking the permutation symmetry of $H_{SE}$ [12]. This requires factoring *Q* into components $Q_{\chi }$ and $Q_{\Theta }$ that act on distinct subsets of qubits and hence distinct sectors of B, i.e., $\mathrm{dom}\left(Q_{\chi }\right)\cap \mathrm{dom}\left(Q_{\Theta }\right)=\emptyset$, with $\mathrm{dom}(Q_{\chi })$ devoted to information exchange and $\mathrm{dom}(Q_{\Theta })$ devoted to TFE exchange. This factorization is advantageous if $\beta_{\Theta }\gg \beta_{\chi }$, with $Q_{\Theta }$ ideally providing all of Ξ(Q) above the Landauer minimum, allowing for the action of $Q_{\chi }$ to minimally disturb *E*. We can represent this situation in schematic form as in Figure 3. It is reflected in the designs of technologies, like transistors, that use separate power inputs and waste-heat outputs to enable high-sensitivity, low-noise computational I/O. It is also evident in the separation between signal transduction and metabolic pathways and between sensory systems and photosynthetic or digestive systems that are observed in biology.

Dividing B into sectors characterized by different thermal efficiencies by functionally distinguishing the sectors $\mathrm{dom}(Q_{\chi })$ or $\mathrm{dom}(Q_{\Theta })$ creates a “difference that makes a difference” [36] in how information flowing through B is processed. Differences between sectors can therefore be thought of as semantic differences—differences effectively in what actions are taken in response to inputs, as well as thermodynamic differences. A choice of a QRF to act on B corresponds, moreover, to a choice of basis vectors for describing both $H_{B}$ and $H_{SE}$ [13]; hence, we can view factorization $Q=Q_{\chi }Q_{\Theta }$ as a choice of distinct representations for the basis vectors characterizing $\mathrm{dom}(Q_{\chi })$ versus $\mathrm{dom}(Q_{\Theta })$. We could, from a mathematical perspective, also choose to maintain constant β and build the energetic difference into a difference between temperatures $T_{\chi }$ and $T_{\Theta }$ associated with $\mathrm{dom}(Q_{\chi })$ and $\mathrm{dom}(Q_{\Theta })$, respectively [37]. Any system that uses a part of its environment with above-average energy density, e.g., external electrical power, solar radiation, or sugar, as a thermal resource effectively takes this approach to the energy/information tradeoff. Organisms typically employ both variable β and variable *T* strategies, e.g., by absorbing relatively high-temperature TFE resources from the environment through specialized anatomical structures with non-uniform bioenergetic properties.

## 4. Measuring and Controlling Energy Usage

Unlike technologies designed for an environment with effectively unlimited energy resources, living systems are often faced with energy scarcity. Restrictions on the availability of TFE are effectively restrictions on computational throughput, rendering the allocation of energy an important “control knob” on computation. It is for this reason that energy usage and its control are significant practical issues for modeling AIAs.

Energy-supply restrictions can prevent a system that has multiple available QRFs from deploying them simultaneously to measure and act on its environment. Deploying multiple QRFs sequentially requires a control system that allocates TFE resources to one QRF at a time. In the context of the FEP, attentional control—how much either a top-down or a bottom-up signal is amplified or attentuated—is standardly modeled as precision adjustment [38,39]. Low-resolution signals can be amplified, and hence have high precision [40], for example, when reflexive attention is driven by the magnocellular visual pathway, which sacrifices object-identification accuracy for speed [41]. Recognizing specific objects as having high significance, e.g., specific individual humans that must be correctly identified, requires both high precision and high resolution, and therefore more bits and more TFE. Hence, attention as precision control can, when high object-identification accuracy is required, automatically control TFE allocation as well; the utility of the blood oxygen level-dependent (BOLD) signal for indicating areas in enhanced neural activity via functional MRI provides striking evidence for this [42]. Targeting energy resources to one QRF at the expense of others requires walling it off, with an interaction-minimizing boundary, from any others that might compete with it. Serialization of QRFs, in other words, induces compartmentalization even of QRFs that would otherwise commute. Hence, systems that are driven by TFE restrictions to deploy QRFs in sequence must be composites of multiple atomic systems, one for each serially deployable QRFs. The converse is also true:

**Theorem** **2.** *Only composite systems can control thermodynamic free energy flows.*

**Proof.**  Since it is clear that composite systems can control TFE flows, it suffices to show that atomic systems cannot. This, however, is obvious: for atomic system *S* to be well defined, its QRF *Q* must be well defined as a computation, and hence have well-defined values for all the terms in Equation (2). □

On a deeper level, Theorem 2 follows from the inability of any system *S* to measure its own boundary; for proof, see ([32], Thm.1, Clause 1).

We suppose now that *S* is a compartmentalized system interacting with an energetically restricted environment *E*. Provided that TFE availability varies slowly compared to the timescale for other inputs from *E*, natural selection processes favor architectures for *S* that include a metaprocessor component *M* that allocates energy resources to *m* other components $S_{1},\cdots S_{m}$ of *S*, each of which can be regarded as atomic [43]. The boundary of *M* must include *m* disjoint sectors $M_{i}$ that each interface with the thermodynamic sector $\Theta_{i}$ of one of the $S_{i}$; these sectors must be disjoint for the boundaries and hence the state spaces of the $\Theta_{i}$ to be well defined. The boundary of *M* must also include a sector that manages its own thermodynamic I/O, i.e., that obtains TFE specifically from and dissipates waste heat specifically into *E*. Each of the $M_{i}$ has an associated QRF, which, to save notation, we can also call $M_{i}$. We assume these QRFs $M_{i}$ all mutually commute, so that *M* can measure the thermodynamic states of, and supply energy to, multiple of the $S_{i}$ simultaneously. No generality is lost with this assumption by taking *M* to be atomic, as any finite hierarchy of metaprocessors must have some top level with this characteristic. Theorem 2 therefore applies to *M*: while *M* can control TFE flows to the $S_{i}$, it cannot address its own energy supply versus computation tradeoff.

We can now ask: how effectively can *M* control the overall computational behavior of *S* by differentially allocating TFE resources to $S_{i}$? The answer clearly depends on *M*’s ability to determine both the need for a particular $S_{i}$ in the current behavioral context and that the resource needs, relative to the rest of *S*, of that $S_{i}$. This information must be obtained from *M*’s environment $E_{M}$, which comprises *E* together with all of the $S_{i}$. Indeed, *M* is just an AIA operating in $E_{M}$.

To recognize that *M* is an AIA operating in $E_{M}$ is, however, to recognize the difference and prima facie mismatch between *M*’s task in the context of *S* and *M*’s task in its own environment, i.e., in $E_{M}$. The former task is effectively to increase *S*’s predictive power, while the latter is to increase *M*’s predictive power (i.e., the task stipulated by *M*’s compliance with the FEP). Compatibility between these tasks requires, at minimum, preventing competition between *M* and the $S_{i}$. The only architecture for *S* that does this is one in which *M* is the sole energetic interface between the $S_{i}$ and *E*, and the $S_{i}$ are collectively the sole informational interface between *M* and *E*. To observed this, note that if $S_{i}$ can obtain TFE independently of *M*, *M* is less able to control their operation to prevent competition or deadlock, and hence less able to optimize *S*’s behavior, while if *M* can obtain information from *E* independently of the $S_{i}$, the FEP drives *M* to optimize its own access to the affordances of *E* instead of optimizing *S*’s access.

This architecture explicitly restricts *M*’s information about *S*’s current behavioral context to that provided by its interaction with the $S_{i}$. The only learnable predictive model for *M* is, therefore, a model of how energy distribution to the $S_{i}$ correlates with expected future energy availability to *M*. The role of *M* in increasing *S*’s predictive power is therefore limited to increasing *S*’s ability to predict future energy availability. This, as mentioned earlier, solves the dark room problem for *S*. It also places an energetic constraint on epistemic foraging that does not positively correlate with energetic foraging. From an organismal perspective, this constraint makes sense; novel information may be very valuable, but its value can only be realized if the energy required to exploit it can also be found. Attention, in other words, is automatically prioritized toward maintaining TFE resources, i.e., to maintaining allostasis. Semelparous species violate this rule, prioritizing sex over TFE, but pay the price when allostasis collapses.

## 5. Resource Usage in the Matrix Representation

As noted earlier, computational simulations of AIAs have tended to ignore energy usage and hence the use of energy allocation as a control knob for system behavior. The discrete matrix formulation of active inference developed in [14] is a general and commonly used tool for such simulations. For present purposes, the most important matrices are A, representing the mapping at some time $t_{i}$ from an internal “belief” state to a predicted observation, and B, representing the time-propagator for internal belief states. In the simplest case in which the only action is belief updating, these are d×d matrices for some fixed dimension *d* in some orthonormal computational basis, i.e., A=Id maps each belief deterministically to a distinct, specific observation and B=Id propagates each belief forward in time unchanged.

We can think of these matrices in either of the ways discussed in Section 2 above. If dimension d=N, corresponding to “observations” of the entire MB B, then A and B describe the entire AIA *S*. If, on the other hand, d<N, corresponding to observations of just some informative sector χ of B, A and B describe a particular inferential process implemented by *S*. This latter interpretation is implicit in Ref. [14], and in simulations that do not take energy usage into account. As the matrix elements have no intrinsic semantics, we could also think of A and B as describing TFE processing alone, or as performing some subprocess with both inferential and thermodynamic components.

If d<N, and we treat the B matrix as encoding inference, we can write an effective N×N matrix B as

(3) $$B=\left[\begin{array}{cc} B_{ij}^{\prime } & b_{ij}^{\prime }\\ b_{ij} & B_{ij} \end{array}\right]$$

where $B_{ij}^{\prime }$ represents the thermodynamic action of B, analogous to the QRF component $Q_{\Theta }$ in Figure 3, and $b_{ij}$ and $b_{ij}^{\prime }$ represent the thermodynamic coupling into, and out of, respectively, the inferential process represented by the d×d matrix $B_{ij}$. This matrix B propagates both belief states and their energy usage forward through time. The A matrix, and any other matrices representing thermodynamically irreversible computations within a given model, can be similarly extended, with analogous interpretations.

If we assume constant *T* and hence $\beta_{\Theta }>\beta_{\chi }$ discussed in Section 3 above, extending a normalized computational basis chosen for B to a basis for B either renders the larger basis unnormalized or requires renormalization to account for net energy flows. From Equation (2), renormalization by $f_{\Theta }\left(n_{\Theta }\right)\beta_{\Theta }/f_{\chi }\left(n_{\chi }\right)\beta_{\chi }$ is required to take differences in the extent of classical computation and hence the amount of TFE that is required between $Q_{\Theta }$ and $Q_{\chi }$ into account.

## 6. Conclusions

What counts as “information processing” by system *S* is observer-relative [19]. The FEP provides the formalism needed to represent the energy dependence of information processing that is a fundamental consequence of embodiment, but this energy dependence is often abstracted out in practice. Keeping it in the model allows addressing control-theoretic issues that cannot be explicitly formulated otherwise.

Modeling energy dependence explicitly helps to emphasize the four-way tradeoff faced by any AIA: the need to balance (1) its requirements for new information (i.e., unpredicted environmental behavior), (2) memory for old information (i.e., predicted results of past actions), and (3) fuel to fund the computing and encoding of these data against (4) the size of its boundary and hence against the risks to boundary integrity posed by a stronger interaction with its environment. “Precarious” or “edge-of-chaos” behavior results when an AIA drives close its the upper limit of boundary-preserving interaction with its environment. Such precarious behavior maximally exposes the AIA to its environment to gain boundary space for both data and fuel, but also maximizes the risk of boundary collapse, failure of allostasis, and death. We can expect that systems for which fuel resources are rare and hard to obtain, e.g., carnivores, and systems that preferentially engage in epistemic foraging, e.g., explorers of new territories, to be forced into this high-risk lifestyle. Sedentary systems for which energetic resources are highly predictable, e.g., photosynthesizing plants in a stable climate, can be expected to adopt a more passive, low-risk lifestyle. Similarly, a big brain and hence a high-energy budget are required by any system that can identify and interact specifically with a large variety of environmental objects, while systems that do not notice or respond to many details of their environments do not have this requirement. Making the thermodynamic cost of being an AIA explicit thus not only helps us understand individual AIAs, but also opens the door to understanding ecologies of AIAs.

## Data Availability

All data are contained in the paper.

## Abbreviations

The following abbreviations are used in this manuscript:

AIA	Active Inference Agent
CCCD	Cone-CoCone Diagram
FEP	Free Energy Principle
QRF	Quantum Refence Frame
TFE	Thermodynamic Free Energy
TQFT	Topological Quantum Field Theory
VFE	Variational Free Energy
